# Single nucleotide polymorphisms in the apolipoprotein B and low density lipoprotein receptor genes affect response to antihypertensive treatment

**DOI:** 10.1186/1471-2261-4-16

**Published:** 2004-09-28

**Authors:** Ulrika Liljedahl, Lars Lind, Lisa Kurland, Lars Berglund, Thomas Kahan, Ann-Christine Syvänen

**Affiliations:** 1 Department of Medical Sciences, Uppsala University, Uppsala University Hospital, Entrance 70, 3^rd ^floor, 751 85 Uppsala, Sweden; 2 Uppsala Clinical Research Center (UCR), Uppsala University, 751 85 Uppsala, Sweden; 3 Division of Internal Medicine, Karolinska Institute, Danderyd Hospital, 182 88 Stockholm, Sweden; 4 Astra Zeneca Research & Development Mölndal, 431 83 Mölndal, Sweden

**Keywords:** Antihypertensive treatment, pharmacogenetics, lipids, minisequencing, genotyping

## Abstract

**Background:**

Dyslipidemia has been associated with hypertension. The present study explored if polymorphisms in genes encoding proteins in lipid metabolism could be used as predictors for the individual response to antihypertensive treatment.

**Methods:**

Ten single nucleotide polymorphisms (SNP) in genes related to lipid metabolism were analysed by a microarray based minisequencing system in DNA samples from ninety-seven hypertensive subjects randomised to treatment with either 150 mg of the angiotensin II type 1 receptor blocker irbesartan or 50 mg of the β_1_-adrenergic receptor blocker atenolol for twelve weeks.

**Results:**

The reduction in blood pressure was similar in both treatment groups. The SNP C711T in the apolipoprotein B gene was associated with the blood pressure response to irbesartan with an average reduction of 19 mmHg in the individuals carrying the C-allele, but not to atenolol. The C16730T polymorphism in the low density lipoprotein receptor gene predicted the change in systolic blood pressure in the atenolol group with an average reduction of 14 mmHg in the individuals carrying the C-allele.

**Conclusions:**

Polymorphisms in genes encoding proteins in the lipid metabolism are associated with the response to antihypertensive treatment in a drug specific pattern. These results highlight the potential use of pharmacogenetics as a guide for individualised antihypertensive treatment, and also the role of lipids in blood pressure control.

## Background

Hypertension is a complex trait caused by multiple environmental and genetic factors interacting through the cardiac, vascular and endothelial systems. Several drug classes with different mechanisms of action, including inhibitors of the renin-angiotensin-aldosterone system (RAAS), calcium channel blockers, adrenergic receptor blockers and diuretics, are available for treatment of hypertension. However, the response to antihypertensive treatment is highly variable between individuals, which makes it difficult to predict the efficacy of a specific drug in the individual patient [1-3]. Currently, there are no clinically useful biochemical or metabolic markers for predicting the individual responses to antihypertensive treatment [4-6].

Twin studies have estimated that as much as half of the variability in blood pressure levels between individuals is due to genetic factors [7,8]. Based on the abundance of single nucleotide polymorphisms (SNPs) in the human genome [9], it can be expected that one or more SNPs occur in each of the genes encoding components of the blood pressure regulating systems, and that they are the genetic factors influencing individual blood pressure levels. Coding SNPs affecting the function of enzymes and receptors in pathways of blood pressure regulation, or regulatory SNPs, affecting the expression levels of genes, are likely to explain part of the variability of the response to antihypertensive treatment. Hence, these functional SNPs, or other SNPs inherited in linkage disequilibrium with them, could be potential pharmacogenetic markers for predicting the response to a certain drug, and thus guide the selection of the optimal drug for each individual patient [10-12].

The RAAS and the sympathetic nervous system play key roles in blood pressure regulation. We have earlier shown that polymorphisms in the angiotensin converting enzyme gene [13] and a SNP in the aldosterone synthase gene [14] are related to changes in blood pressure during treatment with the angiotensin II receptor blocker irbesartan, whereas two SNPs in the angiotensinogen gene were associated to the reduction in blood pressure by the β_1_-adrenergic receptor blocker atenolol [15]. Dyslipidemia with high levels of serum triglycerides and free fatty acids, and elevated serum cholesterol levels and low levels of high-density lipoprotein cholesterol are common in hypertensive patients. Association has been found between disturbance in lipid metabolism and hypertension, but so far no attempts have been made to relate variables reflecting lipids, or the genes involved in lipid metabolism, to the individual response to antihypertensive treatment.

We have recently developed a microarray based minisequencing system for parallel genotyping of multiple SNPs in blood pressure regulating candidate genes [16]. Here we analysed the relationships between the genotypes of SNPs in the apolipoprotein A-IV, apolipoprotein A-V, apolipoprotein B-100, low density lipoprotein receptor, hepatic lipase and lipoprotein genes and reductions in blood pressure in hypertensive patients randomised to monotherapy with either irbesartan or atenolol. We found that SNP alleles in the apolipoprotein B gene and the low density lipoprotein receptor gene were associated to the antihypertensive response after twelve weeks of treatment.

## Methods

### Study population

DNA extracted from blood samples from 97 hypertensive patients from the double blind parallel group "Swedish Irbesartan Left Ventricular Hypertrophy Investigation versus Atenolol" (SILVHIA) trial [17] were analysed. Men and women above the age of 18, having primary mild to moderate hypertension and left ventricular hypertrophy were enrolled in the trial and randomised to receive either 150 mg of the angiotensin II type 1 receptor blocker irbesartan or 50 mg of the β_1_-adrenergic receptor blocker atenolol once daily as monotherapy. The dose was doubled after six weeks if the diastolic blood pressure was ≥ 90 mmHg. Blood pressure was measured by trained nurses using a mercury sphygmomanometer, after the patients had rested for at least 10 min in the seated position. Left ventricular hypertrophy was defined as left ventricular mass index of > 131 g/m^2 ^for men and > 100 g/m^2 ^for women, assessed by echocardiography. The data presented relates to the change in blood pressure after 12 weeks of treatment. For details on the SILVHIA trial, see Malmqvist *etal *[17]. Baseline characteristics for the patients are presented in Table 1. The study was approved by the ethics committees of all participating centres of the SILVHIA trial and that of the Medical Faculty of Uppsala University.

**Table 1:** Characteristics of the hypertensive patients in the two treatment groups.

	**Irbesartan group**^2^	**Atenolol group**^2^
Number of patients	48	49
Age (years)	54 ± 8	54 ± 8
Gender (proportion females)	37%	31%
Height (m)	1.74 ± 0.09	1.73 ± 0.09
Weight (kg)	83 ± 15	82 ± 14
Smokers trial start (%)	29	18
Baseline fs-cholesterol (mM)	6.1 ± 1.0	5.8 ± 1.1
Baseline fb-glucose (mM)	5.7 ± 3.1	5.2 ± 2.5
Pre-treatment SBP^1 ^(mmHg)	164 ± 18	160 ± 20
Pre-treatment DBP^1 ^(mmHg)	104 ± 7	103 ± 8
Change in SBP at 12 weeks (mmHg)	-16 ± 20	-11 ± 16
Change in DBP at 12 weeks (mmHg)	-9.0 ± 11	-12 ± 7.7

^1 ^Systolic blood pressure (SBP) and diastolic blood pressure (DBP)

^2 ^Data are mean ± SD

### SNP markers and genotyping procedure

In our previous study [16], 98 SNPs were selected from the NCBI (dbSNP, ) and the SNP Consortium (TSC, ) databases and validated in a pooled DNA sample representing the Swedish population. A subset of these SNPs located in genes involved in lipid metabolism and that were polymorphic in the Swedish population were included in the study presented here, together with one additional SNP in the apolipoprotein A-V gene. See Table 2 for information on the SNPs.

**Table 2:** Investigated polymorphisms given as gene names, acronym and GenBank accession number.

**Gene name and acronym**^1^	**dbSNP ID**^2^	**Amino acid alteration**	**SNP name**^3^
Apolipoprotein A-IV	rs5092	Thr/Thr	A1449G
APOA-IV; J02758			
Apolipoprotein A-V	rs662799	Promoter	C31455T
APOA-V; AC074203			
Apolipoprotein B-100	rs1801701	Arg/Gln	G10108A^†^
APOB; M19828^†^; M19810^§^	rs1367117	Thr/Ile	C711T^§^
Low density lipoprotein receptor	rs688	Asn/Asn	C16730T
LDLR; AF217403	rs5925	Val/Val	C2000IT
Lipase, hepatic	rs6083	Asn/Ser	A110G
LIPC; M35429			
Lipoprotein	rs328	Ser/Term	C9040G
LPL; AF050163	rs312	Intron	G7315C
	rs314	Intron	A7360G

^1 ^Gene name and acronym, GenBank accession number  for the sequence used in the design of primers for the PCR and minisequencing reactions.

^2 ^SNP identification number in the NCBI SNP database dbSNP, .

^3 ^Corresponding to the nucleotide position in the gene sequence referenced in the first column and the nucleotide variation in the coding strand.

Fragments comprising the SNPs were amplified in multiplex PCR described previously [16]. A microarray based minisequencing single nucleotide primer extension assay, in which one or two of four ddNTPs labelled with the fluorophore Tamra (Perkin Elmer Life Sciences, Boston, MA, USA) were incorporated by the Thermo Sequenase™ DNA-polymerase at each SNP site. The incorporated ddNTPs were detected using a fluorescence scanner, and the fluorescence signals were extracted. A signal intensity fraction, obtained by dividing the fluorescence signal intensity for allele 1 with the sum of the fluorescence signal intensities for allele 1 and allele2, was used to assign the individual genotypes. The SNP APOA-V C31455T was genotyped using a microtiter plate minisequencing assay with tritium detection [18].

### Statistical analyses

Analysis of covariance (ANCOVA) with each SNP as factor, baseline blood pressure as covariate and the change in blood pressure as response, was performed. The analyses were performed by treatment group and blood pressure measurement (systolic and diastolic blood pressures). Correction for multiple testing was performed by calculation of critical p-values corresponding to a nominal type I error of 5% using a permutation test [19]. Two tailed significance levels were used.

## Results and discussion

**Figure 1:**
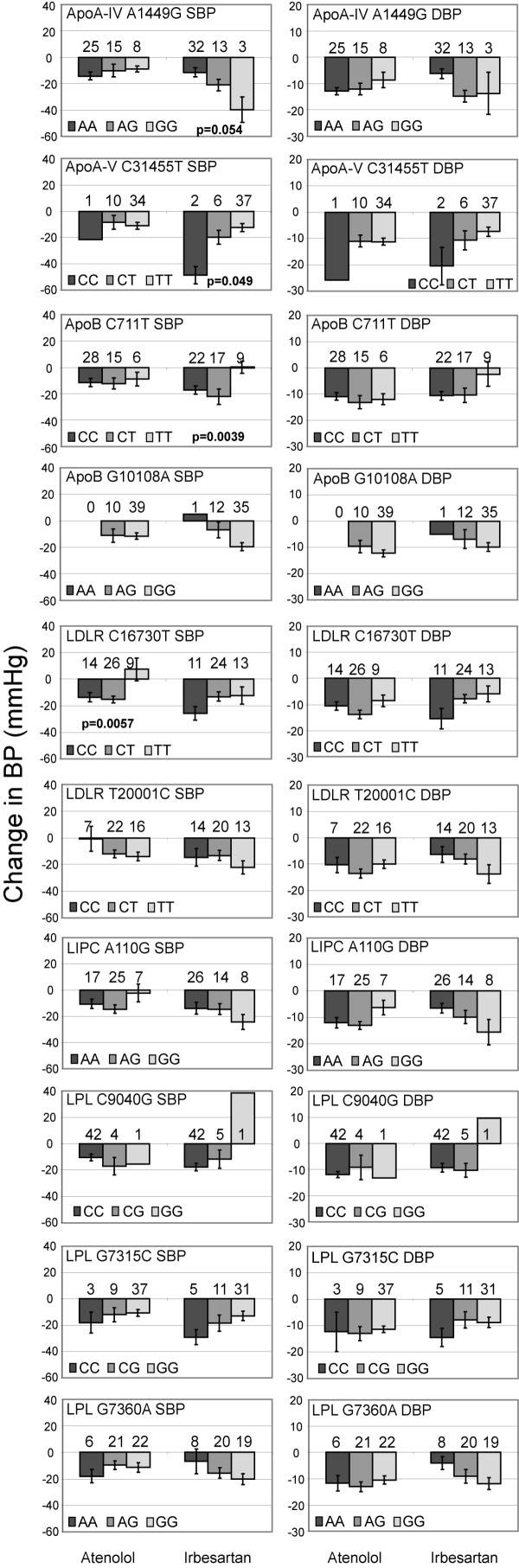
Effect of SNP genotype on the change in blood pressure after 12 weeks of treatment for the ten SNPs. For each of the SNPs, the pattern of change in blood pressure related to genotype is illustrated for the systolic blood pressure (SBP) and diastolic blood pressure (DBP) in separate panels. In each panel, the mean change in blood pressure is shown for the SNP genotypes in the atenolol treatment group on the left, and the corresponding results in the irbesartan treatment group are given on the right. The error bars corresponds to the standard error of the mean. The p-values indicating significance for the APOA-IV A1449G, APOA-V C31455T, APOB C711T and LDLR C16730T SNPs are given in the corresponding panels. The number of individuals of each genotype is shown above the bars in each panel.

We explored possible associations between individual genotypes of ten SNPs and reduction in systolic and diastolic blood pressure as response to treatment with atenolol or irbesartan (Figure 1) in samples from the SILVHIA trial [17]. In the irbesartan group, a change in systolic blood pressure appeared to be related to genotype for the SNPs ApoA-IV A1449G, ApoA-V C31455T and ApoB C711T. In the atenolol treatment group, presence of the C-allele of the SNP LDLR C16730T was associated to the reduction in systolic blood pressure.

Correction for multiple testing using a permutation test [19] yielded critical p-values of 0.004 and 0.007 for systolic blood pressure after atenolol and irbesartan treatment, respectively, and 0.006 and 0.007 for the diastolic blood pressure, corresponding to the significance level of p = 0.05. After the permutation test, carriership of the C-allele of the C711T SNP in the apolipoprotein B gene remained significantly associated to the reduction in systolic blood pressure (p = 0.004) in the irbesartan treatment group (Figure 1) while the individuals homozygous for the T-allele showed no reduction in systolic blood pressure. The same pattern of response related to genotype was seen for diastolic blood pressure, although it did not reach statistical significance. In the atenolol treatment group, the SNP C16730T in the low density lipoprotein receptor gene showed a trend of association to the reduction in systolic blood pressure. Presence of the C-allele was related to blood pressure reduction (p = 0.006), while the individuals homozygous for the T-allele (n = 9) actually showed an increase in systolic blood pressure (Figure 1). A similar response pattern was not seen for the diastolic blood pressure during atenolol treatment (p = 0.44) (Figure 1). There were 39 carriers of the favourable C-allele of the APOB C711T SNP in the irbesartan treatment group. The average reduction in systolic blood pressure for these individuals was 19 mmHg, compared to 0 mmHg for the individuals lacking this allele. In the atenolol treatment group, the individuals carrying the favourable C-allele of the SNP LDLR C16730T showed an average reduction of 14 mmHg in systolic blood pressure compared to an increase of 7.5 mmHg for the individuals homozygous for the T-allele.

The SNP C711T in the apolipoprotein B gene is located in the coding region of the gene, and alters a threonine residue to an isoleucine residue in the protein. This SNP is located in the amino-terminal part of the enzyme and has been suggested to affect the dimerisation of apolipoprotein B and low density lipoprotein during cholesterol transport [20]. The C16730T SNP in the LDLR gene results in a synonymous amino acid change, however this SNP could be in linkage disequilibrium with another functional SNP potentially influencing the response to drug treatment. Irrespectively if the tested SNPs are actually functional, our findings imply a potential connection between lipid metabolism and response to antihypertensive treatment.

We have recently found circulating apolipoprotein B to be the most powerful predictor of endothelium-dependent vasodilation of the commonly used markers of cholesterol metabolism [21]. It is not evident, however, why apolipoproteins predict the response to irbesartan, and not to atenolol treatment, as these drugs appear to improve endothelium-dependent vasodilation to a similar extent [22]. Lipid abnormalities, commonly seen in hypertension, have been considered to be connected to the blood pressure level by the common denominators obesity and insulin resistance. Other studies have suggested a more direct effect of lipids in blood pressure control, as infusion of the fat emulsion Intralipid together with heparin increases blood pressure in healthy subjects [23-25]. This effect is more pronounced in the normotensive subjects with a family history of hypertension [26]. It has also been shown that an acute elevation of free fatty acids alters heart rate variability, an index of cardiac autonomic nervous system balance [27], suggesting that lipid metabolism may be involved in the regulation of cardiovascular autonomic tone. Thus our results that indicate involvement of components of lipid metabolism in the response to antihypertensive treatment are supported by cross-sectional epidemiological studies.

In our earlier exploratory study, 74 SNPs with a minor allele frequency over 5%, including nine of the SNPs analysed here were tested as predictors of blood pressure regulation in the SILVHIA study samples using a multiple regression model [16]. The main aim of this study was to establish the microarray-based genotyping system. Analysis of twenty-eight SNPs from this panel that are located in genes from the renin-angiotensin aldosterone system identified a SNP in the aldosterone synthase gene (CYP11B2 T267C) and two SNPs in the angiotensinogen gene (AGT G1218A and T1198C) that appeared to be associated to blood pressure reduction [14,15]. A limitation in these previous studies was that correction for multiple testing was not applied, whereas in the current study we used a permutation test.

A remaining weakness in our study is the small number of samples available for analysis, which does not allow detection of small to medium size gene effects, and results in uncertain estimation of the the magnitude of the effects detected. Moreover, in a small study there is the risk of a non-representative group of patients with respect to gender, age, and genotype distribution. Despite these limitations, we detected a significant effect of the SNP C711T in the apolipoprotein B gene and the SNP C16730T in the low density lipoprotein receptor after correction for multiple testing. The pharmacogenetically interesting results from our study need to be replicated in other studies.

As the C711T SNP in the apolipoprotein B gene predicted response to treatment with irbesartan, and the C16730T SNP in the low density lipoprotein receptor gene appeared to predict response to atenolol treatment, our results point at possible use of SNPs in genes encoding components of lipid metabolism in pharmacogenetic panels for selecting the optimal drug for each patient. To our knowledge our study is the first one to investigate the relationship between polymorphisms in genes involved in lipid metabolism and the response to antihypertensive treatment.

## Competing interests

The authors declare that they have no competing interests.

## Authors' contributions

UL performed the development of genotyping technology, genotyping lab work, interpretation of data and had a substantial role in writing the manuscript. LL provided clinical expertise, participated in selection of candidate genes and contributed to writing. LK provided clinical expertise, established a database of the SILVHIA phenotypes, and in writing. LB performed the statistical analysis. TK provided the SILVHIA samples and contributed to writing the manuscript. A-CS contributed by planning and supervision of the project, and to writing the manuscript.

## Pre-publication history

The pre-publication history for this paper can be accessed here:


